# A Cross-Sectional Analysis of Young Men’s Gambling and Intimate Partner Violence Perpetration in Mwanza, Tanzania

**DOI:** 10.3389/ijph.2023.1605402

**Published:** 2023-05-19

**Authors:** Rebecca Brambilla, Gerry Hillary Mshana, Neema Mosha, Donati Malibwa, Philip Ayieko, Simon Sichalwe, Saidi Kapiga, Heidi Stöckl

**Affiliations:** ^1^ Institute of Medical Information Processing, Biometry and Epidemiology (IBE), Faculty of Medicine, LMU Munich, Munich, Germany; ^2^ Pettenkofer School of Public Health, Munich, Germany; ^3^ Mwanza Intervention Trials Unit, Mwanza, Tanzania; ^4^ National Institute for Medical Research (Mwanza Centre), Mwanza, Tanzania; ^5^ Department of Infectious Disease Epidemiology, Faculty of Epidemiology and Population Health, London School of Hygiene and Tropical Medicine, University of London, London, United Kingdom; ^6^ Department of Global Health and Development, Faculty of Public Health and Policy, London School of Hygiene and Tropical Medicine, University of London, London, United Kingdom

**Keywords:** young men, intimate partner violence, domestic abuse, gambling, betting

## Abstract

**Objectives:** The prevalence of intimate partner violence (IPV) in Tanzania is one of the highest in sub-Saharan Africa. There are very few studies on the co-occurrence of gambling and IPV and none from LMICs, despite gambling being a behaviour associated with gender norms exalting masculinity underlying IPV perpetration.

**Methods:** Cross-sectional survey data of 755 currently partnered men aged 18–24 from Mwanza, Tanzania were analysed to investigate whether gambling was associated with past-year physical, sexual, emotional and economic IPV. We conducted bivariate and multivariate logistic regressions to control for potential confounders, based on their significant association bivariately with the main outcome variables.

**Results:** Of the men who gambled, 18 percent perpetrated physical IPV, 39 percent sexual IPV, 60 percent emotional IPV and 39 percent economic IPV. Gambling was significantly associated with sexual (aOR: 2.59; 95% CI: 1.70–3.97), emotional (aOR: 1.55; 95% CI: 1.12–2.14) and economic IPV (aOR: 1.38; 95% CI: 1.02–1.88) after controlling for confounders.

**Conclusion:** The analysis shows that gambling is associated with IPV perpetration. More research is needed to understand how current IPV prevention efforts can be expanded to include problem gambling treatment.

## Introduction

Intimate partner violence (IPV) encompasses behaviours of a physical, sexual, and psychologically harmful nature in a relationship, as well as emotional and economic abuse and controlling behaviours [1]. IPV persistently remains one of the greatest global health concerns for women and girls worldwide. The most recent estimates show that globally, 27% of ever-partnered women have experienced physical or sexual violence, or both, in their lifetimes [1].

Tanzania has one of the highest rates of IPV in the World Health Organisation (WHO) African region. The 2015–16 Demographic and Health Survey in the country found that 39% of ever-partnered women aged 15–49 experienced physical, 14% sexual and 36% emotional IPV [2]. The same pattern holds for past-year prevalence, with 24% of Tanzanian women having been subjected to physical and/or sexual IPV in the previous 12 months—the global average being 13% [1]. These figures are high not just compared to global estimates, but also when considering regional ones for sub-Saharan Africa (namely 33% lifetime prevalence and 20% past-year prevalence [1]).

Several studies have examined various potential risk factors for IPV, from socioeconomic determinants [2–4] to health-related factors, such as poor mental health and addiction [5–8], with some identifying social norms [9–12] and traditional gender roles [13–16] as key determinants of both IPV perpetration and victimisation. An increasing focus in recent years on factors concomitant with IPV perpetration in men has highlighted how socially-constructed gender norms that exalt traditional masculinity and conventional “manly” behaviours [17–20] like drinking and risk-taking are associated with IPV. Some studies in the Tanzanian context in particular have underscored how alcohol drinking [21] and traditional masculine norms [22–25] in men underlie and trigger IPV perpetration and sexual harassment, with men who feel “emasculated” more likely to resort to violence in order to reassert their power in the family.

In many contexts, gambling (i.e., the practice of betting money in games of chance in the hope of winning something of value in return) is a behaviour usually associated with traditional masculine traits such as being daring and reckless. Given the role traditional masculine norms play in IPV perpetration, it is surprising that there is very little evidence on the association between IPV perpetration and gambling.

Most studies on the co-occurrence of gambling and IPV are set in Australia [26–36], Canada [37], the United Kingdom [38] and the United States [39–42]. The single systematic review and meta-analysis on the topic [43] only includes studies conducted in Australia, Canada, New Zealand, Spain and the United States. To date there is no evidence on this phenomenon coming from low-income countries and from African countries in particular.

The existing studies draw attention to the harmful nature of gambling and gambling addiction in particular, highlighting its strong ties to family violence in general and IPV specifically. Despite using a variety of tools to measure gambling (the South Oaks Gambling Screen (SOGS) [38, 39, 41], the Brief Bio-Social Gambling Screen (BBGS) [26, 27], the Problem Gambling Severity Index (PGSI) [30, 44], the Canadian Problem Gambling Inventory (CPGI) [37], the Victorian Gambling Screen (VGS) [28], the Alcohol Use Disorder and Associated Disability Interview Schedule-DSM-IV Version (AUDADIS-IV) [42], and the Gambling Motivation Questionnaire for Financial Motivations (GMQ-F) [45, 46]), virtually all studies show an association between gambling and one or multiple forms of IPV. Qualitative studies also corroborate these findings, illustrating the various difficulties family members of gamblers face, from experiencing threats and intimidation, control and manipulation, relationship conflict and violent outbursts, to dealing with economic exploitation and financial problems [31, 33, 34, 36].

Some of the existing studies have hypothesised that, whereas the causal and temporal link between gambling and IPV perpetration remains uncertain, gambling-associated stressors can intensify IPV by exacerbating conflicts within the couple [36, 42]. Another reason frequently mentioned for the co-occurrence of IPV perpetration and gambling is poor impulse control [30, 38, 39, 41, 47]. Relatedly, most studies cite mental disorders, alcohol dependence, drug and substance abuse, and aggression as risk factors for both gambling and IPV perpetration [27, 28, 32, 34, 37, 38, 40, 41]. Interestingly, several studies mention that a strong risk of experiencing gambling-related harm persists also for less severe and non-problem gamblers [34, 38, 40, 46].

**FIGURE 1:**
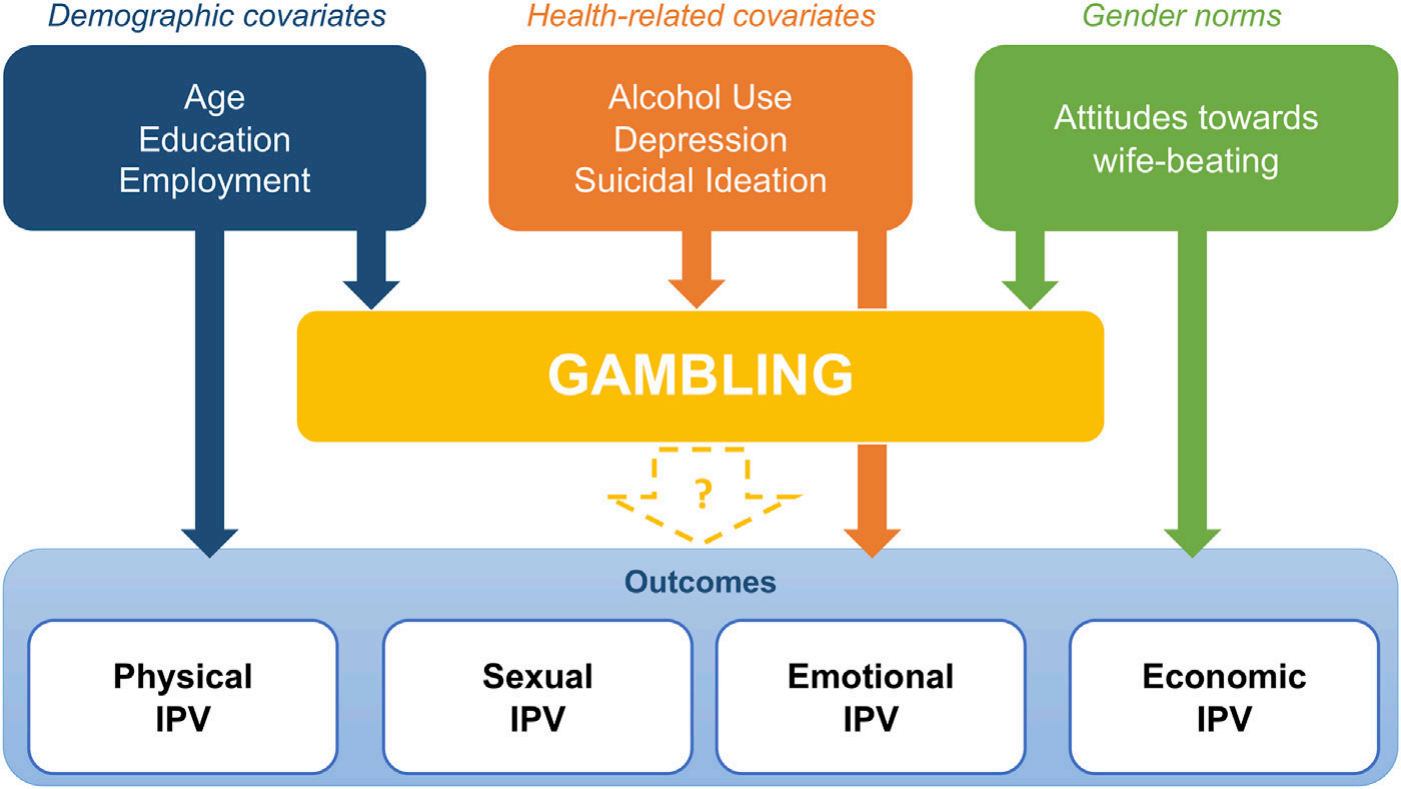
Conceptual framework of the association between gambling and intimate partner violence perpetration (MAISHA study, Tanzania, 2021–2022).

The issue with most of the existing studies is that they conflate different forms of IPV (physical, sexual, emotional and economic) in one single indicator, instead of looking at them separately [43]. By doing so, they muddle the different pathways that can lead from gambling to IPV. Figure 1 summarizes potential confounders for the association of gambling and IPV.

### Gambling in Sub-Saharan Africa

Despite a paucity of research on gambling disorder in the sub-Saharan African (SSA) context, Ssewanyana and Bitanihirwe [48] estimate that 54% of youth have engaged in some form of gambling activity. The results of this study are echoed by Ahaibwe et al., who found that in Kampala “the youth (18–30 years) are more likely to engage in gambling compared to their older counterparts (31 years and above)” ([49]: 7). Similarly, Kiwujja et al. [50] report that 62% of their sample of people aged 15–24 in Kampala disclosed gambling. These findings are particularly concerning, considering the African continent has the youngest population in the world, with 70% of SSA under the age of 30 [51].

Young males were identified as more likely to engage in gambling-related activities, compared to young females [48–50, 52], which is explained by young women generally being more risk-averse [29, 52]. This patterns holds in studies conducted outside the SSA context, with Hing et al. [29] finding that in their Victoria, Australia sample, problem gambling was twice as likely in men compared to women, and risk factors included being aged 18–24 years old. Moreover, in a recent qualitative study of men aged 22–42 conducted in Mwanza, Tanzania [53], gambling emerged as a recurrent topic that warranted worry for the influence it has on young men.

In Tanzania there are both legal and illegal forms of gambling. The former, commonly defined by the Kiswahili word “Kamari,” can take place in different formats not dissimilar to those available in most HICs. The most prevalent ones in the country are sports betting, particularly on football matches, betting through FM Radio stations and TV stations, and lotteries. These are common among most strata of the population, and especially popular among young men. Slot machines are also legal and they are found mostly in bars and arcades in places where there is a high population density. Casinos with different types of games and betting machines are also present in the country and are visited by patrons of higher socioeconomic status.

Illegal forms of gambling take place mainly in the streets, with gamblers often playing with cards or dice. These forms of gambling are outlawed due to their connection to theft and other petty crime, and engagement in illegal gambling is believed to be connected to substance abuse.

To date, no study has investigated the association between gambling and IPV in the SSA context. To resolve the above-mentioned gaps, the current study aims to analyse the relationship between gambling and IPV perpetration in a sample of young Tanzanian men aged 18–24, as well as potential associations with other known risk factors, with the aim of better understanding how different forms of IPV perpetration are associated with gambling.

## Methods

### Sample

Between June 2021 and April 2022, a cross-sectional survey with 1,002 men aged 18 to 24 was conducted in Mwanza, Tanzania. Six wards were selected from a shortlist of 13 in the Illemela and Nyamagana districts. A stratified random selection of wards was conducted within the two districts and two strata of population destiny, aiming to include three densely populated and three sparsely populated wards. A random sample of 24 streets was selected across the six wards in Illemela district (three wards) and Nyamagana district (three wards).

The team worked together with street leaders to pinpoint street boundaries for mapping and get introduced to households within the community, in order to identify potential participants that fit the survey criteria (male aged 18 to 24, who had lived in the area for longer than 3 months). For each street, 120 points were randomly generated and the two closest households to each point were visited to identify eligible participants. Only one young man was randomly selected from each household.

The team visited a total of 2,976 households: 1,065 of these had young men meeting the eligibility criteria stated above and 1,911 did not. If more than one young man in the chosen household met our survey criteria, random selection of survey participants was conducted by having a family member randomly pick one of the names of young men living in the household that were written on folded paper. The procedure was done openly and transparently as to ensure that all potential candidates had equal chances of being selected.

Seven young men declined to participate in the study and fourteen had agreed to take part during the sampling exercise but were not reachable or available during the survey period. Another 42 men were subsequently excluded for other reasons (i.e., having moved away Mwanza).

Trained male fieldworkers interviewed all participants who provided informed consent and administered a structured questionnaire. Whereas general demographic, household and health questions were asked face-to-face, sensitive questions on violence were asked via headphones and answered by the participants on a tablet.

Informed consent was obtained from all participants. Ethical approval for this study was granted by the National Health Research Ethics Committee in Tanzania, the London School of Hygiene and Tropical Medicine and the Ludwig Maximilian University in Munich.

### Survey Measures

Perpetration of physical, sexual, emotional and economic IPV were assessed through acts-based questions with the answer categories “yes” and “no” taken from the IMAGES study [54] and Sonke CHANGE trial [55], which include questions from the UN Multi-country Cross-sectional study on Men and Violence [56]. Both sets of perpetration questions were validated among men in different cultural settings to capture male perpetration of IPV. The questionnaire asked if the participant had ever perpetrated a specific act against a partner and whether this had happened in the past 12 months, to assess both lifetime and past year prevalence. This analysis will focus on past year prevalence of each form of IPV.

Past year economic, physical and sexual violence were measured with three yes/no questions each, which were combined into a single indicator for every form of violence (answering “yes” to at least one question = 1, answering “no” to all three questions = 0). As part of the physical IPV questions, men were asked if they had ever slapped, pushed or shoved a female partner [56]. Sexual violence questions asked for example, if the participant had forced a partner to have sex [55]. Taking a partner’s earnings against her will was one of the questions assessing economic IPV [56]. Past year emotional violence was measured with six yes/no questions, which were combined into a single indicator (answering “yes” to at least one questions = 1, answering “no” to all six questions = 0). Examples of emotional IPV questions include having insulted, belittled or humiliated a partner [55]. The full list of questions used to assess IPV perpetration can be found in the Supplementary Material.

The gambling questions used in this survey were informed by existing survey tools developed in high-income countries [57–59] and adapted to the local context after extensive pilot testing of the questionnaire, resulting in five yes/no questions that were further amended based on evidence from qualitative research conducted in the same setting, in order to measure participation in legal gambling activities in the previous year. The Swahili word “Kamari” was used in the questionnaire, to imply legal forms of gambling versus illegal gambling. This was done to avoid the risk of underreporting of gambling habits by study participants, and also to build trust with the community and not give the impression the fieldworkers were investigating any illegal activities.

The first question asks whether the participant had bet or spent money on gambling or gambling machines in the past 12 months, and was used to measure the dependent variable; the other four assess consequences of gambling, for instance if the participant has lied to family members to hide gambling or whether gambling has caused any health problems, but were left out of the analysis. The list of questions used to assess gambling and consequences thereof can be found in the Supplementary Material.

### Covariates

Covariates usually associated with IPV and/or gambling were also measured as part of the study. Socioeconomic covariates included: age; education (no education, at least primary, at least secondary, college and university); employment status (yes, no) and employment type (employed, self-employed). Health-related covariates included: depressive symptoms (measured through the PHQ-9 questionnaire [60], categorised as none/minimal, mild, moderate to severe); suicide ideation/attempt (measured through questions from the CoVAC study [61], yes, no); alcohol use (measured through the AUDIT score [62], categorised as abstainer, low-risk consumption, harmful alcohol consumption, alcohol dependent) and drug use (yes, no). One gender attitude-related covariate was measured through a questions asking whether it could be justified for a man to beat his wife (never, sometimes, always, do not know).

### Data Analysis

The data analysis was conducted using STATA 17.0 and accounting for potential clustering of IPV outcomes within the 24 streets that were sampled. After analysing descriptive characteristics of the sample, cross-tabulations and Mantel-Haenszel tests were performed to examine the relationship between the exposure variable (gambling) and four outcome variables (physical, sexual, emotional and economic IPV) separately, as well as the above-listed covariates. In the following stage, binary logistic regressions were used to determine the strength of the association between the potential confounders and each of the outcomes. The variables that were statistically significantly associated with IPV (*p* < 0.05) in the bivariate analyses were added to the logistic regression model for that particular type of IPV, alongside all socioeconomic variables (age, education and employment) irrespective of whether these were significantly associated with IPV in the bivariate analyses.

## Results

### Descriptive Statistics

Out of 1,002 young men interviewed, 755 said to have been in a relationship in the previous 12 months. The first two columns of Table 1 show the characteristics of this sample. 76% of the men have been employed in the previous year (N = 574), with roughly half describing their employment as self-employed.

**TABLE 1:** Background characteristics of the sample and associations between covariates and intimate partner violence perpetration (N = 755) (MAISHA study, Tanzania, 2021–2022).

	N	%	Physical violence (N = 124 (16.42))			Sexual violence (N = 176 (23.13))			Emotional violence (N = 357 (47.28))			Economic violence (N = 231 (30.60))		
			n/N (%)	Crude OR (95% CI)	*p*-value	n/N (%)	Crude OR (95% CI)	*p*-value	n/N (%)	Crude OR (95% CI)	*p*-value	n/N (%)	Crude OR (95% CI)	*p*-value
Age														
18	87	11.52	18/87 (20.69)			17/87 (19.54)			40/87 (45.98)			26/87 (29.89)		
19	112	14.83	19/112 (16.96)			26/112 (32.21)			50/112 (44.64)			35/112 (31.25)		
20	99	13.11	18/99 (18.18)			15/99 (15.15)			42/99 (42.42)			25/99 (25.25)		
21	146	19.34	26/146 (17.81)			36/146 (24.66)			66/146 (45.21)			48/146 (32.88)		
22	91	12.05	8/91 (8.79)			21/91 (23.08)			42/91 (46.15)			27/91 (29.67)		
23	113	14.97	17/113 (15.04)			35/113 (30.97)			53/113 (46.90)			33/113 (29.20)		
24	107	14.17	18/107 (16.82)	0.94 (0.86–1.03)	0.203	26/107 (24.30)	1.08 (0.97–1.19)	0.144	64/107 (59.81)	1.08 (1.00–1.16)	0.061	37/107 (34.58)	1.02 (0.93–1.12)	0.617
Education														
No education	88	11.66	23/88 (26.14)	1		19/88 (21.59)	1		36/88 (40.91)	1		25/88 (28.41)	1	
At least primary	287	38.01	52/287 (18.12)	0.62 (0.37–1.06)	0.081	72/287 (25.09)	1.22 (0.64–2.30)	0.547	133/287 (46.34)	1.25 (0.81–1.92)	0.318	90/287 (31.36)	1.15 (0.66–2.01)	0.621
At least secondary	296	39.21	40/296 (13.51)	0.44 (0.26–0.75)	0.003	62/296 (20.95)	0.96 (0.48–1.94)	0.915	151/296 (51.01)	1.50 (0.91–2.49)	0.113	92/296 (31.08)	1.14 (0.62–2.07)	0.675
College and university	84	11.12	9/84 (10.71)	0.34 (0.12–0.91)	0.032	23/84 (27.38)	1.37 (0.60–3.14)	0.458	37/84 (44.05)	1.14 (0.54–2.37)	0.732	24/84 (28.57)	1.01 (0.52–1.94)	0.981
Employment														
Yes	574	76.03	100/574 (17.42)	1.38 (0.83–2.28)	0.209	142/574 (24.74)	1.42 (0.96–2.10)	0.079	273/574 (47.56)	1.05 (0.77–1.42)	0.766	187/574 (32.58)	1.50 (0.92–2.45)	0.100
No	181	23.97	24/181 (13.26)	1		34/181 (18.78)	1		84/181 (46.41)	1		44/181 (24.31)	1	
Employment type														
Employed	286	49.83	52/286 (18.18)	1.11 (0.70–1.77)	0.658	77/286 (26.92)	1.26 (0.92–1.73)	0.146	143/286 (50.00)	1.21 (0.87–1.70)	0.256	98/286 (34.27)	1.16 (0.78–1.73)	0.450
Self-employed	288	50.17	48/288 (16.67)	1		65/288 (22.57)	1		128/288 (45.14)	1		89/288 (30.90)	1	
Depressive Symptoms														
None/Minimal	445	58.94	64/445 (14.38)	1		71/445 (15.96)	1		177/444 (39.78)	1		119/445 (26.74)	1	
Mild	243	32.19	45/243 (18.52)	1.35 (0.80–2.28)	0.258	84/243 (34.57)	2.78 (1.97–3.93)	<0.0001	133/243 (54.73)	1.83 (1.31–2.56)	<0.0001	82/243 (33.72)	1.39 (1.06–1.84)	0.018
Moderate to severe	67	8.87	15/67 (22.39)	1.72 (0.82–3.59)	0.151	21/67 (31.34)	2.40 (1.44–4.03)	0.001	47/67 (70.15)	3.56 (2.22–5.70)	<0.0001	30/67 (44.78)	2.22 (1.47–3.35)	<0.0001
Suicide ideation/attempt														
Yes	42	5.56	7/42 (16.67)	1.02 (0.41–2.50)	0.968	17/42 (40.48)	2.37 (1.21–4.63)	0.012	28/42 (66.67)	2.33 (1.32–4.12)	0.004	19/42 (45.24)	1.95 (1.26–3.03)	0.003
No	713	94.44	117/713 (16.41)	1		159/713 (22.30)	1		329/713 (46.14)	1		212/713 (29.73)	1	
Alcohol use														
Abstainer	566	74.97	76/566 (13.43)	1		117/566 (20.67)	1		249/566 (43.99)	1		155/566 (27.39)	1	
Low risk consumption	96	12.72	14/96 (14.58)	1.10 (0.48–2.54)	0.822	22/96 (22.92)	1.14 (0.79–1.64)	0.476	45/96 (46.88)	1.12 (0.69–1.82)	0.636	33/96 (34.38)	1.39 (0.88–2.20)	0.162
Harmful alcohol consumption	66	8.74	25/66 (37.88)	3.93 (1.99–7.76)	<0.0001	25/66 (37.88)	2.34 (1.38–4.00)	0.002	48/66 (72.73)	3.39 (2.13–5.42)	<0.0001	29/66 (43.94)	2.08 (1.40–3.07)	<0.0001
Alcohol dependent	27	3.58	9/27 (33.33)	3.22 (1.37–7.59)	0.007	12/27 (44.44)	3.07 (1.15–8.18)	0.025	15/27 (55.56)	1.59 (0.71–3.54)	0.254	14/27 (51.85)	2.85 (1.49–5.47)	0.002
Drug use														
No	722	95.63	116/722 (16.07)	1		168/722 (23.27)	1		336/722 (46.54)	1		220/722 (30.47)	1	
Yes	33	4.37	8/33 (24.24)	1.67 (0.81–3.45)	0.165	8/33 (24.24)	1.05 (0.40–2.79)	0.914	21/33 (63.64)	2.01 (0.94–4.28)	0.070	11/33 (33.33)	1.14 (0.58–2.23)	0.700
Wife-beating attitudes														
Never justified	579	76.79	81/579 (13.99)	1		125/579 (21.59)	1		261/579 (45.08)	1		176/579 (30.40)	1	
Sometimes justified	147	19.50	34/147 (23.13)	1.85 (1.28–2.67)	0.001	40/147 (27.21)	1.36 (0.92–1.99)	0.119	80/147 (54.42)	1.45 (1.02–2.08)	0.040	46/147 (31.29)	1.04 (0.69–1.58)	0.843
Always justified	28	3.71	9/28 (32.14)	2.91 (1.52–5.59)	0.001	11/28 (39.29)	2.35 (0.94–5.88)	0.068	15/28 (53.57)	1.40 (0.64–3.07)	0.393	9/29 (32.14)	1.08 (0.57–2.06)	0.804

n, number of men who committed IPV, N, total of men in the sample, OR, Odds Ratio, CI, Confidence Interval.

More than half of the sample (59%) did not report having depressive symptoms, but 32% disclosed having some mild and 9% having moderate to severe symptoms. Just under 6% of the participants admitted to having had suicidal thoughts or having made a suicide attempt in their lives. Only 25% of the sample admitted to having drunk an alcoholic beverage in the previous year. Of these, 13% are considered low-risk consumers, 9% harmful alcohol consumers and 4% being alcohol dependent. Nearly 96% of the sample never used any form of drug. Just under a fourth of the sample (23%) found wife-beating to be always or sometimes justified.

Of the 755 men in a relationship, 181 (24%) report having spent money on gambling in the past year. Of these, 25% has bet more than they could afford to lose (N = 46); 50% have spent more money than they wanted on gambling (N = 91); 51% have lied to family members or others to hide their gambling (N = 92) and 62% report gambling having caused them health problems, including stress and anxiety (N = 112).

About 16% of the participants report having committed physical IPV (N = 124), 23% had perpetrated sexual IPV (N = 176), 47% emotional IPV (N = 357) and 31% economic IPV (N = 231) (Table 1, top row).

Physical IPV decreased with increasing education level and was significantly associated with harmful alcohol consumption and alcohol dependency (Table 1). It was also associated with the belief it was sometimes or always justified for a man to beat one’s wife. Sexual IPV perpetration was significantly associated with displaying depressive symptoms and with suicidal ideation, as well as harmful alcohol consumption and alcohol dependency.

Emotional IPV was strongly associated with having depressive symptoms, with suicidal ideation, and with harmful alcohol consumption (but not with alcohol dependency). Finally, economic IPV was associated with having depressive symptoms and suicidal ideation. It was also strongly associated with harmful alcohol consumption and alcohol dependency (Table 1).

### Cross-Tabulations and Mantel-Haenszel Tests

As shown in Table 2, 18% of the men who gambled in the previous 12 months admitted to having committed physical IPV against a partner (N = 32); 39% reported sexual IPV (N = 71); 60% reported emotional IPV(N = 109) and 39% related economic IPV (N = 71).

**TABLE 2:** Association between gambling and intimate partner violence forms adjusted for age (MAISHA study, Tanzania, 2021–2022).

Risk factor	n/N (%)	Adjusted OR (95% CI)*	*p*-value
Physical violence			
Has gambled (past 12 months)			
No	92/574 (16.03)	1	
Yes	32/181 (17.68)	1.12 (0.65–1.93)	0.673
Sexual violence			
Has gambled (past 12 months)			
No	105/574 (18.29)	1	
Yes	71/181 (39.23)	2.90 (1.96–4.28)	<0.0001
Emotional violence			
Has gambled (past 12 months)			
No	248/574 (43.21)	1	
Yes	109/181 (60.22)	2.00 (1.46–2.74)	<0.0001
Economic violence			
Has gambled (past 12 months)			
No	160/574 (27.87)	1	
Yes	71/181 (39.23)	1.67 (1.20–2.33)	0.002

n, number of men who committed IPV, N, total of men in the sample, OR, Odds Ratio, CI, Confidence Interval.

*adjusted for age.

After adjusting for age, gambling was statistically significantly associated with sexual IPV perpetration (aOR: 2.90, 95% CI: 1.96–4.28, *p* < 0.0001), with emotional IPV (aOR: 2.00, 95% CI: 1.46–2.74, *p* < 0.0001) and with economic IPV (aOR: 1.67, 95% CI: 1.20–2.33, *p* = 0.002). The association between gambling and physical IPV was not statistically significant.

### Regression Analyses and Models

As displayed in Table 3, after controlling for socioeconomic covariates, as well as alcohol use, depressive symptoms and suicidal ideation, sexual IPV perpetration remains statistically significantly associated with gambling (aOR: 2.59, 95% CI: 1.70–3.97, *p* < 0.0001). The same is true for both emotional IPV (aOR: 1.55, 95% CI: 1.12–2.14, *p* = 0.007) and economic IPV (aOR: 1.38, 95% CI: 1.02–1.88, *p* = 0.038), which are still associated with gambling after controlling for those same confounders.

**TABLE 3:** Adjusted estimates of the OR for perpetration of intimate partner violence (MAISHA study, Tanzania, 2021–2022).

Risk factor	n/N (%)	Adjusted OR (95% CI)	*p*-value
Physical violence^a^			
Has gambled (past 12 months)			
No	92/574 (16.03)	1	
Yes	32/181 (17.68)	0.95 (0.55–1.64)	0.862
Sexual violence^b^			
Has gambled (past 12 months)			
No	105/574 (18.29)	1	
Yes	71/181 (39.23)	2.59 (1.70–3.97)	<0.0001
Emotional violence^c^			
Has gambled (past 12 months)			
No	248/574 (43.21)	1	
Yes	109/181 (60.22)	1.55 (1.12–2.14)	0.007
Economic violence^d^			
Has gambled (past 12 months)			
No	160/574 (27.87)	1	
Yes	71/181 (39.23)	1.38 (1.02–1.88)	0.038

n, number of men who committed IPV, N, total of men in the sample, OR, Odds Ratio, CI, Confidence Interval.

^a^ Adjusted for age, alcohol use, education, employment.

^b^ Adjusted for age, alcohol use, depressive symptoms, suicidal ideation, education, employment.

^c^ Adjusted for age, alcohol use, depressive symptoms, suicidal ideation, education, employment.

^d^ Adjusted for age, alcohol use, depressive symptoms, suicidal ideation, education, employment.

In the final model physical IPV perpetration remains not statistically significantly associated with gambling (aOR: 0.95, 95% CI: 0.55–1.64, *p* = 0.862).

Holding positive attitudes towards wife-beating was associated with physical and emotional IPV, but did not change the OR when added to the respective models, and was therefore left out of the final model.

## Discussion

This study is one of the first in sub-Saharan Africa linking gambling to IPV perpetration. After adjusting for potential confounders, past-year sexual, emotional and economic IPV perpetration remain associated with gambling. Interestingly, physical IPV did not show an association with gambling in either the crude or adjusted analysis.

This enquiry mirrors the findings of previous studies [34, 38, 40, 46] that gambling in itself, regardless of severity, poses a risk for adverse outcomes and related harm. It also corroborates that, as with most existing literature from studies conducted in other continents [28, 32, 34, 37, 38, 40, 41], alcohol use, depressive symptoms and suicidal ideation moderate part of the relationship between gambling and the outcomes.

Moreover, the high prevalence and strong association between gambling and emotional IPV in the sample can be explained as gambling losses causing increased stress in the perpetrators as well as intra-household tensions, which result in arguments and subsequent emotional abuse [34, 35]. Hing et al. [34] also underscore how emotional violence in the form of verbal abuse is systematically used by gamblers to silence criticism of their gambling, and often also to coerce their partners to subside to their demands to provide more money for gambling.

This evidence also resonates with previous findings—especially from qualitative studies [33–35]—which claim that financial abuse is at the core of the IPV experience of women partnered with gamblers. Since our study is based on self-reports by men, interviewing female partners of gamblers in this context might also help shed light on whether they too feel financially abused by their gambling partners.

The strong association between sexual IPV and gambling has also been reported elsewhere [30, 34, 37, 43]. Indeed, in their analysis of psychological, physical and sexual aggression, Brasfield et al. [41] find that lifetime gambling was uniquely associated with the perpetration of sexually aggressive behaviour among abusive partners only, even after controlling for known confounders.

This particular finding could also provide an explanation as to why no association was found in our study between physical IPV and gambling. Most existing studies on IPV perpetration and gambling use only one generic question which combines physical and sexual (and sometimes emotional/psychological) IPV, conflating all forms of IPV into a single item [43]. It therefore remains unknown whether most gamblers reporting IPV perpetration in those studies do so by admitting to sexual coercion or emotional abuse alone.

Our findings are critical for devising successful programmes to reduce IPV, which should include addressing gambling as both a symptom of traditional masculine norms [23] and a factor that introduces or increases conflict within a household and leads to IPV. Teaching couples negotiation and conflict resolution skills that consider financial pressures from gambling might be an effective way to decrease several forms of IPV. Addressing gambling and gambling addiction will simultaneously foster mental wellbeing in the population.

### Strengths and Limitations

The novel contribution of this study is having explored the association between gambling and IPV perpetration in the Tanzanian setting, whereas most studies on the subject have been conducted in high-income countries. Investigating gambling and its adverse consequences in a sample of young Tanzanian men is especially important, considering the proven high prevalence of the phenomenon in a younger, male population. A further contribution of this study is having looked at different forms of IPV separately, contrary to most existing studies which create a single indicator for IPV perpetration. This allowed us to isolate sexual, emotional and economic IPV as positively associated with gambling.

One key limitation of this study is its cross-sectional nature, which makes it impossible to determine temporality and causality in the association between gambling and IPV perpetration. The study also relies on retrospective self-reports of IPV and gambling by study participants, as well as other antisocial behaviours such as drinking and drug use, which might have been affected by social desirability bias and stigmatization, and therefore led to an underestimation of the true prevalence of some of these phenomena. However, we believe underreporting of IPV perpetration was substantially reduced by participants self-administering the questions on violence through tablets.

Secondly, some risk factors other studies investigated [37, 38, 42], which could affect both gambling behaviour and IPV perpetration—namely impulse control and propensity for risk-taking—were not included in our survey questionnaire. Questions from the Structured Clinical Interview for DSM-IV Personality Disorders Screening Questionnaire (SCID-II) were successfully used by Roberts et al. [38] to capture the presence of impulsivity in violent men with gambling problems, whereas Korman et al. [37] employed the State-Trait Anger Expression Inventory-II (STAXI-II) to understand whether the presence of clinically significant anger problems increased the likelihood of IPV perpetration in their sample of problem gamblers. Future studies on the co-occurrence of gambling and IPV perpetration would benefit from capturing both these variables and including them in their analyses, to have a more all-encompassing view of personality-related risk factors.

Moreover, the study did not use a validated instrument to measure gambling, as no validated instrument has yet been developed for the Tanzanian context, which could have affected the ability to correctly classify the sample. At the same time, the questions on gambling were tailored to the local population after piloting the survey, making them perhaps more valid and reliable than using standardised measures from studies conducted in high-income settings. It is worth noting that the questionnaire used did not capture the frequency of gambling, which might be positively correlated with more frequent and severe forms of IPV perpetration.

As for the generalisability of this analysis, it would be useful to conduct similar studies in other parts of Tanzania and SSA, to address the critical lack of research on the subject in these settings, to develop a validated tool for the context, as well as to corroborate the outcomes of this study.

### Conclusion and Recommendations

Understanding all potential risk factors for IPV is crucial to curb the incidence of this phenomenon, in SSA and globally. So far, gambling has remained vastly under-researched as key factor increasing the odds of IPV perpetration, especially in low-income countries. This study shows a significant association of gambling with sexual, emotional and economic IPV perpetration, which is partly explained by depressive symptoms, suicidal ideation, and alcohol use. Further studies investigating the association of gambling with other known risk factors for IPV could strengthen both prevention and response efforts to this phenomenon.

## Supplementary Material

Supplementary Table S1 Unadjusted associations between gambling and covariates (N = 755) (MAISHA study, Tanzania, 2021–2022).

Supplementary Table S2 Survey questions on Intimate Partner Violence perpetration (MAISHA study, Tanzania, 2021–2022).

Supplementary Table S3 Survey questions on gambling and consequences (MAISHA study, Tanzania, 2021–2022).
