# Supplementary material for: A Cross-Sectional Analysis of Young Men’s Gambling and Intimate Partner Violence Perpetration in Mwanza, Tanzania
Source: Int J Public Health. 2023 May 19;68:1605402. doi: 10.3389/ijph.2023.1605402 (PMC10235485; doi:10.3389/ijph.2023.1605402)
Supplement: Supplementary file 1 [file DataSheet1.pdf]

Table S1: Unadjusted associations between gambling and covariates (N=755) (MAISHA study, Tanzania, 2021-2022)

Covariates	n / N (%)	Crude OR (95% CI)	p-value
Age			
18	22/87 (25.29)		
19	30/112 (26.79)		
20	20/99 (20.20)		
21	34/146 (23.29)		
22	19/91 (20.88)		
23	30/113 (26.55)		
24	26/107 (24.30)	0.99 (0.90-1.10)	0.939
Education			
No education	9/88 (10.23)	1	
At least primary	58/287 (20.21)	2.22 (1.13-4.36)	0.020
At least secondary	91/296 (30.74)	3.90 (1.87-8.09)	<0.0001
College and university	23/84 (27.38)	3.31 (1.51-7.26)	0.003
Employment			
Yes	134/574 (23.34)	1	
No	47/181 (25.97)	1.15 (0.68-1.94)	0.597
Employment type			
Employed	74/286 (25.83)	1	
Self-employed	60/288 (20.83)	0.75 (0.52-1.10)	0.142
Depressive Symptoms			
None/Minimal	88/445 (19.78)	1	
Mild	65/243 (26.75)	1.48 (0.97-2.27)	0.072
Moderate to severe	28/67 (41.79)	2.91 (1.76-4.81)	<0.0001
Suicide ideation/attempt			
No	163/713 (22.86)	1	
Yes	18/42 (42.86)	2.53 (1.41-4.53)	0.002
Alcohol use			
Abstainer	108/566 (19.08)	1	
Low risk consumption	30/96 (31.25)	1.93 (1.23-3.02)	0.004
Harmful alcohol consumption	30/66 (45.45)	3.53 (2.36-5.28)	<0.0001
Alcohol dependent	13/27 (48.15)	3.94 (1.85-8.37)	<0.0001

(Table S1 continued)

**Covariates**	**n / N (%)**	**Crude OR (95% CI)**	**p-value**
Drug use			
No	172/722 (23.82)	1	
Yes	9/33 (27.27)	1.20 (0.43-3.36)	0.730
Wife-beating attitudes			
Never justified	137/579 (23.66)	1	
Sometimes justified	38/147 (25.85)	1.12 (0.73-1.72)	0.587
Always justified	5/28 (17.86)	0.70 (0.26-1.91)	0.487

n=number of men who gambled, N=total of men, OR=Odds Ratio, CI=Confidence Interval

Table S2: Survey questions on Intimate Partner Violence perpetration (MAISHA study, Tanzania, 2021-2022)

**Physical Intimate Partner Violence perpetration**	**Answers**
Thinking of any partner you ever had in your whole life, have you ever slapped, pushed or shoved her or thrown something at a partner that could hurt her?	Yes<br>No
Has this happened in the past 12 months?	Yes<br>No
Thinking of any partner you ever had in your whole life, have you ever hit a partner with a fist or something else that could hurt her, kicked, dragged, beaten, choked or burned her?	Yes<br>No
Has this happened in the past 12 months?	Yes<br>No
Thinking of any partner you ever had in your whole life, have you ever threatened to use or actually used a gun, knife or other weapon against a partner?	Yes<br>No
Has this happened in the past 12 months?	Yes<br>No

**Sexual Intimate Partner Violence perpetration**	**Answers**
Thinking of any partner you ever had in your whole life, have you ever forced a partner to have sex with you when she did not want to?	Yes<br>No
Has this happened in the past 12 months?	Yes<br>No
Thinking of any partner you ever had in your whole life, have you ever had sex with a partner when you knew she didn't want it, but you believed she should agree because she was your wife/partner?	Yes<br>No
Has this happened in the past 12 months?	Yes<br>No
Thinking of any partner you ever had in your whole life, have you ever forced a partner to do something sexual that she did not want to?	Yes<br>No
Has this happened in the past 12 months?	Yes<br>No

**Emotional Intimate Partner Violence perpetration**	**Answers**
Thinking of any partner you ever had in your whole life, have you ever spread rumours about her or tried to turn her friends against her?	Yes<br>No
Has this happened in the past 12 months?	Yes<br>No
Thinking of any partner you ever had in your whole life, have you ever spoken to her in a mean (hostile) tone of voice?	Yes<br>No

Has this happened in the past 12 months?	Yes<br>No
Thinking of any partner you ever had in your whole life, have you ever insulted her or deliberately said things to make her feel bad about herself?	Yes<br>No
Has this happened in the past 12 months?	Yes<br>No
Thinking of any partner you ever had in your whole life, have you ever made fun of her, belittled her or humiliated her in front of other people?	Yes<br>No
Has this happened in the past 12 months?	Yes<br>No
Thinking of any partner you ever had in your whole life, have you ever tried to scare or intimidate her on purpose, for example by the way you looked at her, by shouting, or by smashing things?	Yes<br>No
Has this happened in the past 12 months?	Yes<br>No
Thinking of any partner you ever had in your whole life, have you ever threatened to hurt or actually hurt people she cares about as a way of hurting her, or damage things of importance to her?	Yes<br>No
Has this happened in the past 12 months?	Yes<br>No

**Economic Intimate Partner Violence perpetration**	**Answers**
Thinking of any partner you ever had in your whole life, have you ever made important financial decisions without her?	Yes<br>No
Has this happened in the past 12 months?	Yes<br>No
Thinking of any partner you ever had in your whole life, have you ever prohibited her from getting a job, going to work, trading or earning money?	Yes<br>No
Has this happened in the past 12 months?	Yes<br>No
Thinking of any partner you ever had in your whole life, have you ever taken her earnings against her will?	Yes<br>No
Has this happened in the past 12 months?	Yes<br>No

Table S3: Survey questions on gambling and consequences of gambling (MAISHA study, Tanzania, 2021-2022)

**Gambling and consequences questions**	**Answers**
In the past 12 months, have you bet or spent money on gambling or gambling machines?	Yes<br>No
In the past 12 months, have you bet more than you could really afford to lose?	Yes<br>No
In the past 12 months, have you bet or spent more money than you wanted to on gambling?	Yes<br>No
In the past 12 months, have you lied to family members or others to hide your gambling?	Yes<br>No
In the past 12 months, has gambling caused you any health problems, including stress or anxiety?	Yes<br>No
